# Construction of stemness gene score by bulk and single-cell transcriptome to characterize the prognosis of breast cancer

**DOI:** 10.18632/aging.204963

**Published:** 2023-08-18

**Authors:** Jun Lin, Deyi Feng, Jie Liu, Ye Yang, Xujin Wei, Wenqian Lin, Qun Lin

**Affiliations:** 1 Department of Anesthesiology, The First Affiliated Hospital, Fujian Medical University, Fuzhou 350005, China; 2 Department of Anesthesiology, National Regional Medical Center, Binhai Campus of the First Affiliated Hospital, Fujian Medical University, Fuzhou 350212, China; 3 Anesthesiology Research Institute, The First Affiliated Hospital, Fujian Medical University, Fuzhou 350005, China; 4 Xiamen University, Xiamen 361100, China; 5 Department of Endoscopy, Shengli Clinical Medical College of Fujian Medical University, Fuzhou 350001, China; 6 The First Affiliated Hospital of Fujian Medical University, Fuzhou 350005, China; 7 The Graduate School of Fujian Medical University, Fuzhou 350001, China

**Keywords:** breast cancer, prognosis, single-cell RNA-sequencing, tumor microenvironment

## Abstract

Breast cancer (BC) is a heterogeneous disease characterized by significant differences in prognosis and therapy response. Numerous prognostic tools have been developed for breast cancer. Usually these tools are based on bulk RNA-sequencing (RNA-Seq) and ignore tumor heterogeneity. Consequently, the goal of this study was to construct a single-cell level tool for predicting the prognosis of BC patients. In this study, we constructed a stemness-risk gene score (SGS) model based on single-sample gene set enrichment analysis (ssGSEA). Patients were divided into two groups based on the median SGS. Patients with a high SGS scores had a significantly worse prognosis than those with a low SGS, and these groups exhibited differences in several tumor characteristics, such as immune infiltration, gene mutations, and copy number variants. Our results indicate that the SGS is a reliable tool for predicting prognosis and response to immunotherapy in BC patients.

## INTRODUCTION

Breast cancer is the most commonly diagnosed cancer and the leading cause of cancer-related death among females worldwide. According to 2020 worldwide cancer statistics, female breast cancer had surpassed lung cancer as the leading contributor to global cancer incidence; it accounts for an estimated 2.3 million new cases and 685,000-related deaths, representing 11.7% of all cancer cases and 15.5% of cancer-related deaths in females [1–3]. Management of breast cancer is multidisciplinary. It includes locoregional therapy (surgery and radiation therapy) and systemic therapy (endocrine therapy, chemotherapy and immunotherapy). Due to the high heterogeneity of breast cancer, patients with similar clinical characteristics may have different prognoses [4, 5]. This means that a high proportion of patients present with late-stage disease and a poor prognosis. Therefore, it is important to generate robust tools for prognosis prediction and therapeutic response assessment because these would further facilitate precise and individualized treatment.

Rapid advancements in transcriptome sequencing technologies enabled the development of prognostic tools based on gene expression levels in breast cancer [6]. Bulk RNA-Seq, which is based on RNA extracted from tissue homogenates or cells, can only represent the average expression of genes and cannot capture heterogeneity in complex tissues or cell populations [7, 8]. To overcome this limitation, single-cell RNA sequencing (scRNA-seq) technology was proposed by Tang et al. [9]. ScRNA-seq is a powerful tool for characterizing the transcriptomic profile of individual cells. In recent years, significant improvements in the sensitivity and accuracy of scRNA-seq technology have led to a better understanding of tumor heterogeneity [10, 11]. Nevertheless, since scRNA-seq data for patients usually lack prognostic information and the number of patients is limited, a research gap remains in the study of breast cancer prognosis.

Cancer stem cells (CSCs) represent a subpopulation of cancer cells with the ability to self-renew and drive tumor growth, recognized as tumor initiating cells [12]. CSC has a critical role in tumorigenesis, metastasis and resistance to therapy, also been regarded as an attractive target for cancer treatment [13]. Growing evidence showed that breast cancer stem cells (BCSCs) play a pivotal role in breast cancer development and progression [14, 15]. Therefore, a comprehensive understanding of CSCs can significantly improve treatment recommendations and facilitate the development and personalization of targeted therapies for breast cancer.

In this study, we employed ssGSEA based on 36 publicly available stemness gene sets to obtain a comprehensive view of the genetic landscape of breast cancer by analyzing gene expression profiles across multiple datasets. Our results indicated that breast cancer patients with a high SGS had a worse prognosis than those with a low SGS. Further analyses revealed that these patients exhibited significant deviations in PAM50 and immunohistochemistry (IHC) subtypes tended toward the HER2^+^ and TNBC subtypes, both of which are associated with a poor prognosis. Taken together, our findings suggest that the SGS can serve as an independent and reliable prognostic factor for breast cancer.

## RESULTS

### The SGS identifies two subclasses in BRCA

**Figure 1:**
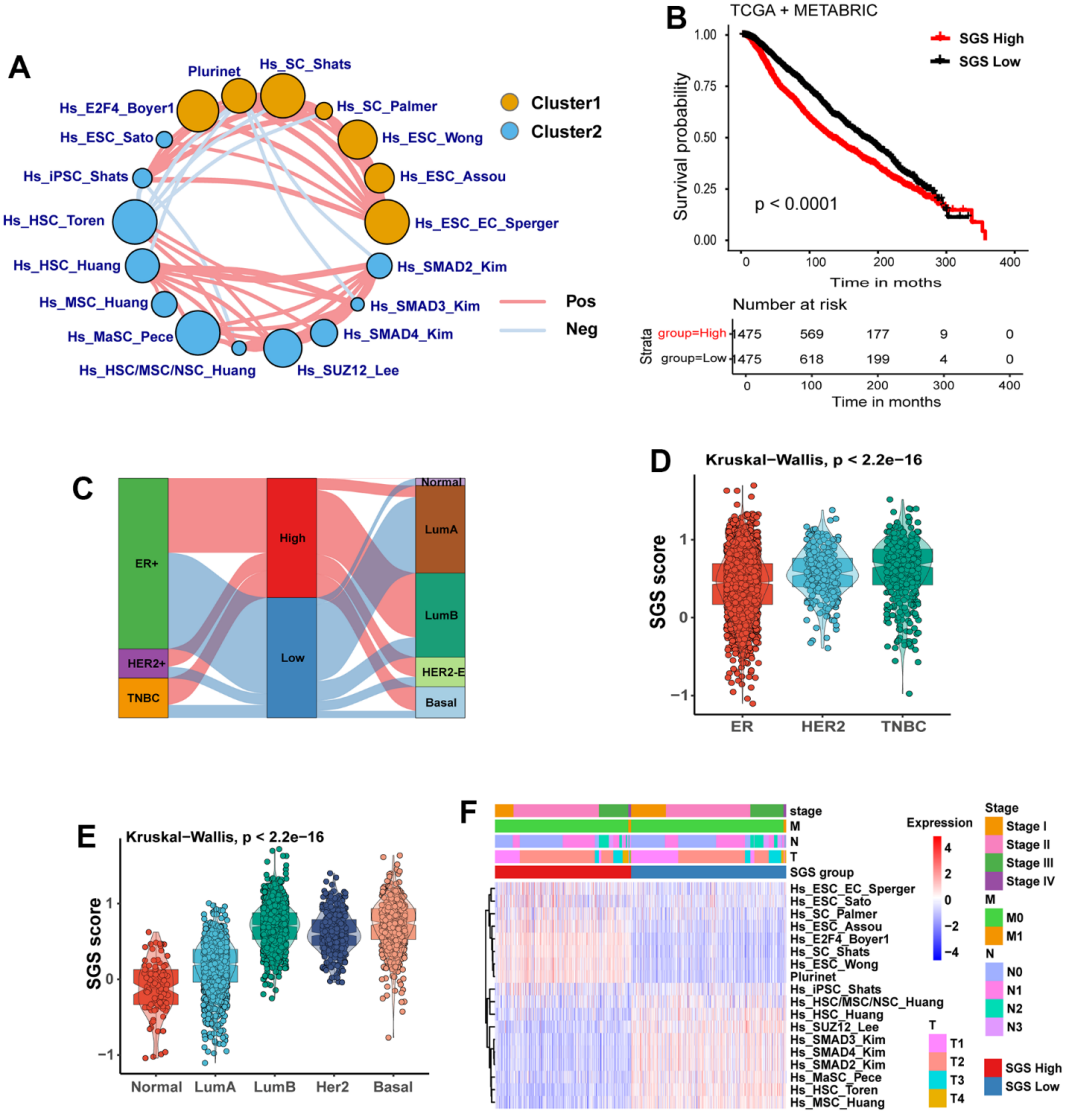
(**A**) Landscape plot of the effect of 18 prognosis-related stem gene sets in breast cancer on survival of BC patients. Cluster 1, orange; cluster 2, blue; HR>1 is Cluster 1, HR<1 is Cluster 2; circle size represents significance. The lines connecting gene sets represent cellular interactions. The thickness of the line represents the strength of correlation. Positive correlation is indicated in red and negative correlation in light blue. (**B**) KM curves for OS of breast cancer patients from TCGA and METABRIC cohort. (**C**) Sankey plot showing the relationship between SGS grouping and IHC typing as well as PAM50 molecular typing. (**D**) Box plot showing the correlation between SGS and IHC subtypes of breast cancer patients. (**E**) Box plot showing the correlation between SGS and PAM50 molecular typing of breast cancer patients. (**F**) Heatmap manifesting the relationship between SGS groupings and clinical pathological parameters in the TCGA.

A flow diagram for the present analysis is shown in Extended Data Supplementary Figure 1. First, we used ssGSEA to calculate the normalized enrichment score (NES) of 36 stemness-related gene sets in the TCGA-BRCA and METABRIC integrated datasets. Then, the prognostic value of these NES values was evaluated by univariate Cox regression analysis. We identified 18 gene sets with the P-value <0.05 as prognostic factors (Supplementary Figure 2A, 2B) and drew a prognostic network of stemness genes (Figure 1A). We calculated he SGS for each patient by subtracting the risk factor score from the protective factor score and stratified all patients into high SGS group and low group by using the median SGS as the cutoff. Kaplan-Meier (KM) survival analysis showed that the high SGS group had a worse prognosis than the low SGS group (Figure 1B).

### Patient characteristics

By analyzing the IHC subtypes of the study population, we found that TNBC and HER2**^+^** patients, two subtypes with a poor prognosis, were more common in the high SGS group. ER**^+^** subtype patients with a better prognosis tended to be in the low SGS group (Figure 1C, 1D). We also observed that among PAM50 classifications of the study population, luminal-B (LumB), HER2-enriched (HER2-E) and basal-like subtypes patients with a poor prognosis were mainly distributed the high SGS group. Luminal-A (LumA) and normal-like subtype patients with better prognosis were mainly distributed in the low SGS group (Figure 1E). Other clinical features, including clinical tumor stage and TNM stage, did not differ significantly between the high SGS group and the low SGS group (Figure 1F).

### Correlation of immune mechanisms with the SGS in breast cancer

**Figure 2:**
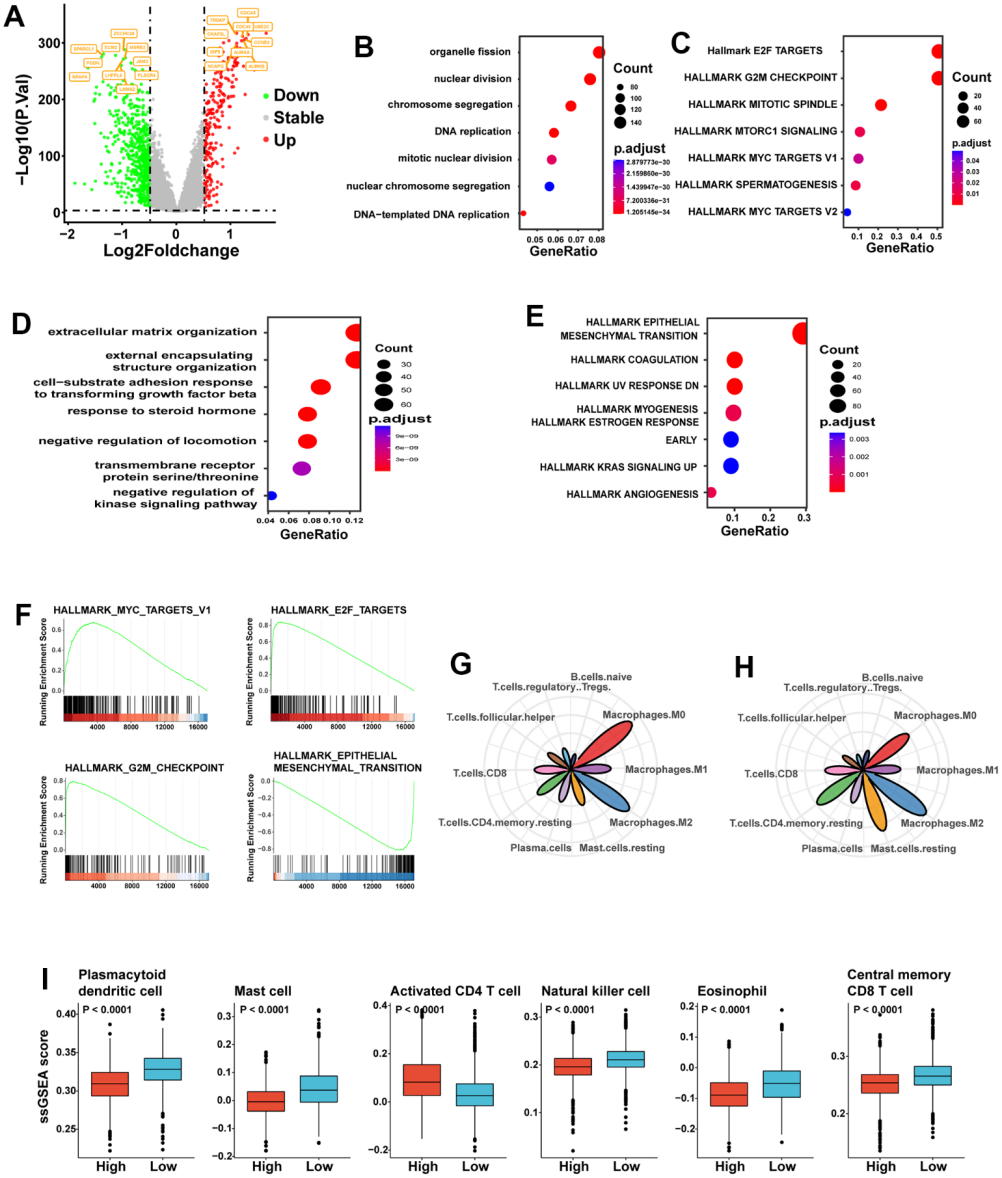
(**A**) Volcano plot of difference analysis between high and low SGS group. Top10 up- and down-regulated genes were tagged. (**B**) GO enrichment analysis of the high SGS group. (**C**) Hallmark gene set enrichment analysis of the high SGS group. (**D**) GO enrichment analysis of the low SGS group. (**E**) Hallmark gene set enrichment analysis of the low SGS group. (**F**) Gene set enrichment analysis of SGS high and low group. (**G**, **H**) Radar charts showing the immune cell infiltration abundances in high (**G**) and low (**H**) stemness-risk groups. (**I**) Boxplot showing differences of some representative immune cells between the high SGS and low SGS group.

To identify mechanisms associated with a poor prognosis, we performed differential gene expression analysis between the two groups. Differentially expressed genes were split into up-regulated and down-regulated genes. Numerous genes associated with the cell cycle, including UBE2C and CCNE2, were up-regulated in the high SGS group (Figure 2A). Then, we performed analysis of up-regulated genes and down-regulated genes separately. We performed functional enrichment analysis on the differentially up-regulated genes using Gene Ontologies (GO) analysis and found that organelle fission, nuclear division and chromosome segregation-related pathways were highly enriched in the high SGS group (Figure 2B). Hallmark gene set enrichment analysis revealed that a series of signaling pathways related to cell proliferation, such as the E2F target, G2M checkpoint and mitotic spindle, were significantly up-regulated in the high SGS group (Figure 2C). GO analysis (Figure 2D) showed that down-regulated genes were mainly enriched in extracellular matrix organization and external encapsulating structure organization-related pathway. Hallmark gene set enrichment analysis showed that down-regulated genes were mainly enriched in epithelial-mesenchymal transition-related pathway. Gene set enrichment analysis (GSEA) analysis indicated similar pathway enrichment (Figure 2F).

We estimated the infiltration of immune cell types using CIBERSORT. We displayed only the 10 immune cells with high content; M0 and M1 macrophages levels were significantly higher in the high SGS group than in the low SGS group, while M2 macrophage levels was slightly less abundant than in the low SGS group. The levels of resting mast cells and resting memory CD4 cells were significantly lower in the high SGS group than in the low SGS group, while the levels of follicular helper T cells (Tfh) and regulatory T cells (Tregs) were higher than in the low SGS group (Figure 2G, 2H). Among the four immune cells with the most significant differences, activated CD4 T cells were more abundant in the high SGS group than in the low SGS group, while mast cells, dendritic cells and central memory CD8 T cells were less abundant in the high SGS group than in the low SGS group (Figure 2I).

### Validation of the SGS model using two independent datasets

**Figure 3:**
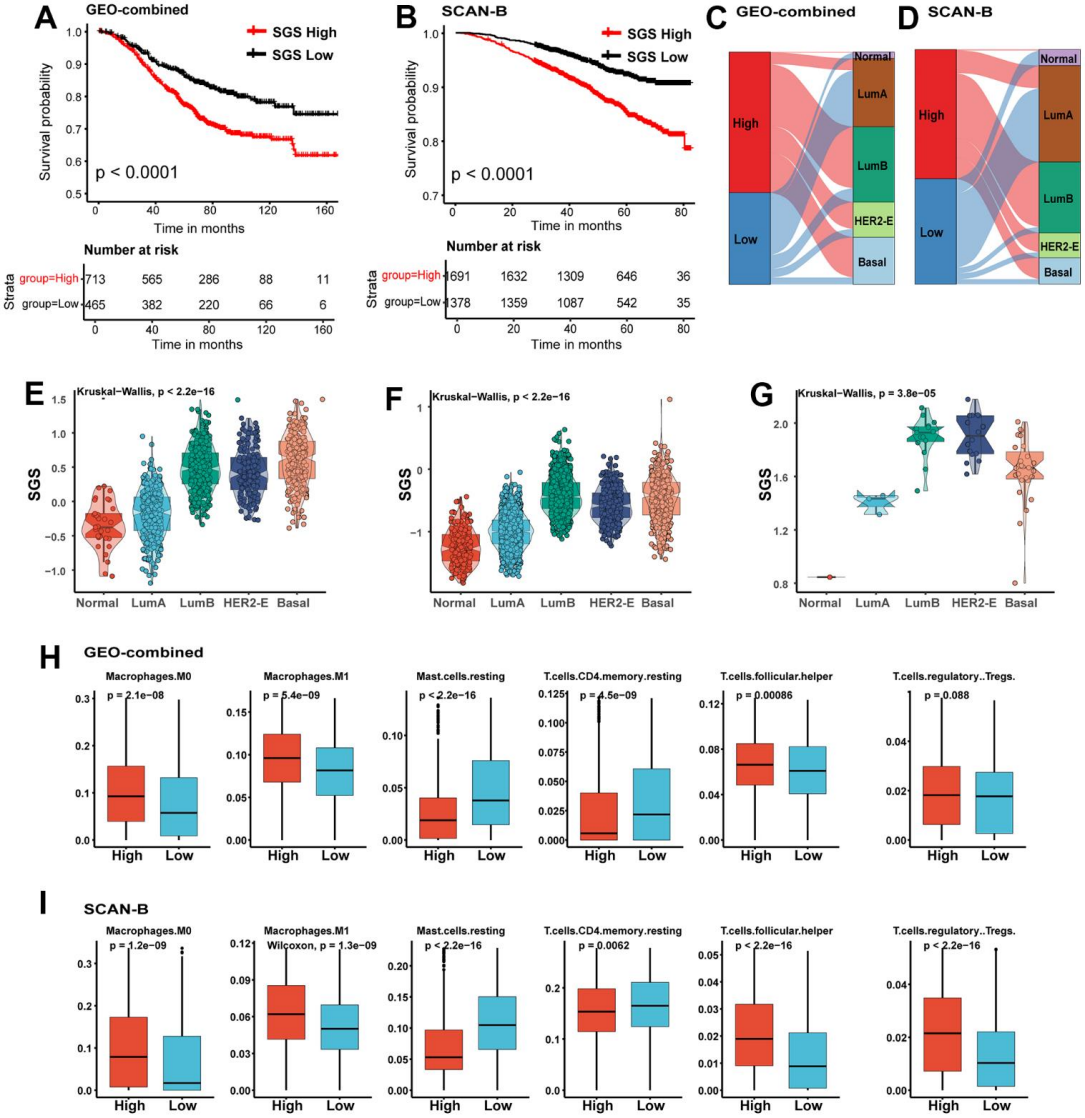
(**A**, **B**) KM curves for OS of BC patients from GEO-combined (**A**) and SCAN-B (**B**) cohort. (**C**, **D**) Sankey plot of PAM50 subtypes in SGS high and SGS low group of GEO-combined (**C**)and SCAN-B (**D**) cohort. (**E**, **F**) Boxplot showing the distribution of SGS among PAM50 subtype in GEO-combined (**E**) and SCAN-B (**F**) cohort. (**G**) Boxplot showing the distribution of SGS among PAM50 subtype of breast cancer cell lines from the CCLE database. (**H**, **I**) Boxplot showing differences of some representative immune cells between SGS high and SGS low group in GEO-combined (**H**) and SCAN-B (**I**) cohort.

To further verify the accuracy of SGS in predicting the prognosis of breast cancer patients, we employed two independent datasets included the SCAN-B dataset and integrated datasets for multiple breast cancer obtained from the GEO database (GEO-combined dataset) as validation datasets. We found that SCAN-B and GEO-combined dataset, breast cancer patients with high SGS also had a poor prognosis (Figure 3A, 3B), and the LumB, HER2-E and basal-like subtypes were the dominant molecular subtypes (Figure 3C, 3D). Similarly, patients with these subtypes also had high SGS (Figure 3E, 3F). We also calculated SGS for breast cancer cell lines from the Cancer Cell Line Encyclopedia (CCLE) database. LumB, HER2-E and basal-like type breast cancer cell lines had a higher SGS (Figure 3G). Immune infiltration analysis also showed that the content of macrophage, Tfh and Tregs in the high SGS group in the validation set was comparable to that in the test set (Figure 3H, 3I).

The immune infiltration analysis also indicated that the majority of immune cells in the high and low SGS groups had the same trend in SCAN-B and GEO-combined dataset as the training set (Supplementary Figure 3A–3C). The differentially expressed genes in the test set also had a very strong correlation with prognosis in these two validation sets (Supplementary Figure 3D, 4E). All these results indicate that the SGS has a strong generalizability and is applicable for different sequencing methods and different sources of data.

### Correlation of gene mutations with the SGS in breast cancer

**Figure 4:**
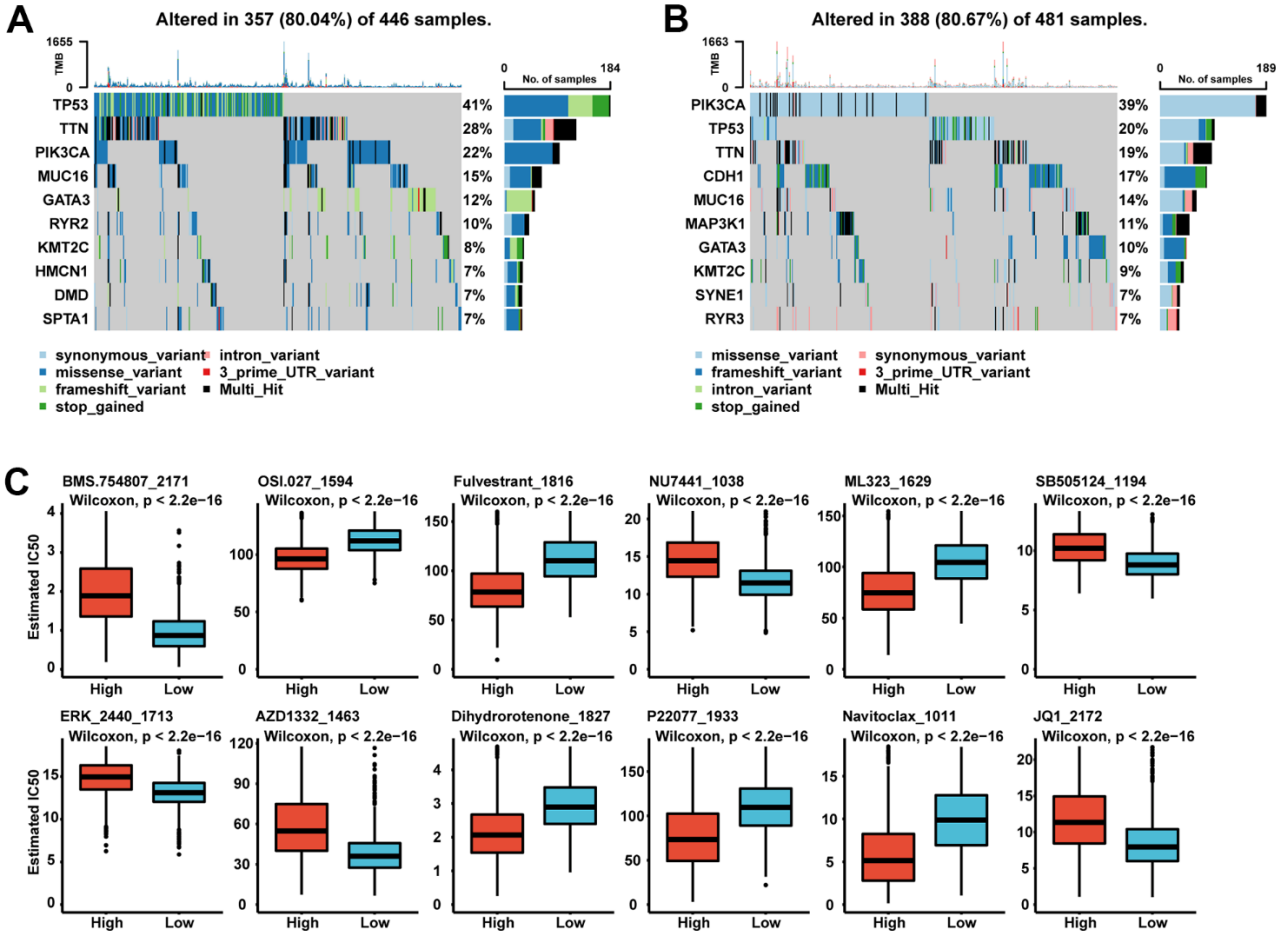
(**A**, **B**) Oncoplot of landscape of mutation signatures between high SGS group (**A**) and low SGS group (**B**) in TCGA. (**C**) Boxplot of some representative of predicted IC50.

Therefore, we wanted to investigate whether there were differences in gene mutations between patients in the SGS high and low group. Although the two groups had approximately 80% of mutations observed in all genes, the two groups differed in the pattern of gene mutations. P53 and PI3KCA are the most frequently mutated genes in breast cancer. The high SGS group had a higher P53 mutations rate, while the low SGS group had a higher PI3KCA mutation rate. TTN gene mutations were also more common in patients in the high SGS group than in those in the low SGS group. For RYR family genes, the high SGS group was more likely to have RYR2 gene mutations, while the low SGS group was more likely to have RYR3 gene mutations (Figure 4A, 4B).

### The SGS could predict therapeutic benefit

Currently, chemotherapy is still the mainstay treatment for most BC patients, so we performed IC50 prediction of common chemotherapy drugs for patients. We found significant differences in sensitivity to chemotherapeutic agents in patients with different SGS subgroups (Figure 4C). We found lower IC50 scores for osimertinib (OSI), fluvestrant and navitoclax in the SGS high group, which means that these chemotherapy drugs have significant clinical efficacy in patients with a high SGS. The results suggest that we can predict sensitivity to targeted therapies, but further study is required.

### SGS application for scRNA-seq data

**Figure 5:**
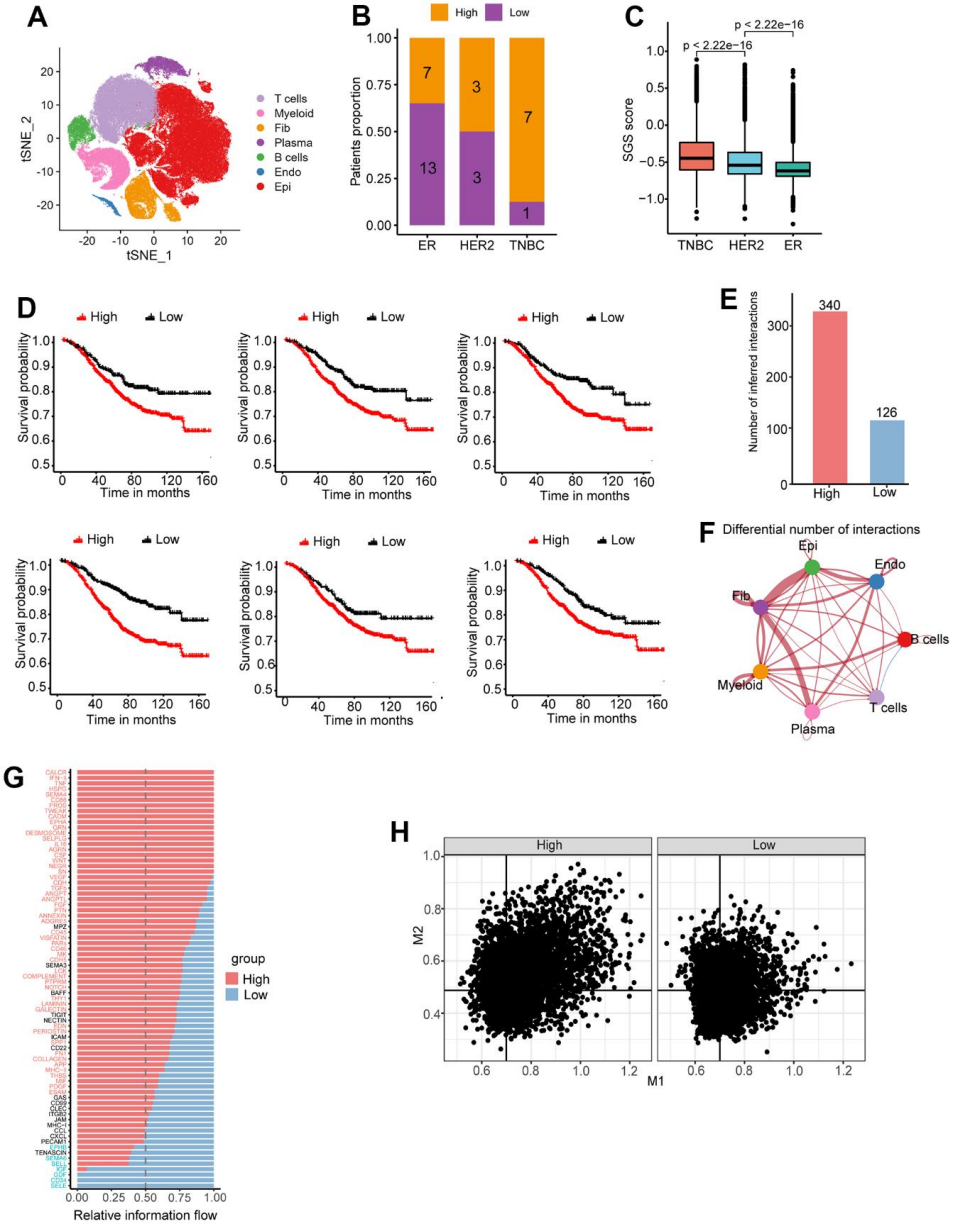
(**A**) Cluster analysis and dimension reduction used non-linear dimensional reduction (t-SNE) in breast cancer scRNA-seq. (**B**) Bar graph of patient proportion (SGS high/SGS low) with different immunohistochemical typing. (**C**) Boxplot showing distribution of SGS among different IHC typing. (**D**) KM curves of top6 differential genes for high and low SGS groups of the SCAN-B cohort. (**E**) Number of intercellular interactions for high and low SGS groups in breast cancer scRNA-seq. (**F**) A landscape plot of intercellular interactions strengths differences compared SGS high group to SGS low group. Red represents positive correlation and blue represents negative correlation. The thickness of the lines represents the degree of difference. (**G**) Bar plot of overall information flow of some signaling pathway between high and low SGS group. (**H**) Scatter plot of M1 and M2 signature gene score of per macrophage in SGS high and low patients. Black lines indicate median scores of characteristic genes.

ScRNA-seq is a new technique that allows transcriptome analysis of individual cells. We selected the largest single-cell data cohort of breast carcinoma *in situ* newly published in 2021 [16]; it contains data for a total of 20 ER^+^, 6 HER2^+^ and 8 TNBC breast cancer patients. After data integration and cluster analysis, major cell clusters, including epithelial cells, immune cells, fibroblasts and endothelial cells, were identified (Figure 5A). We computed the SGS value for each epithelial cell, calculated the mean SGS of each patient, and divided patients into high and low SGS groups based on the median SGS. Seven of the eight TNBC-type patients were classified in the high SGS group (Figure 5B). Overall, the SGS of epithelial cells in TNBC-type patients were greater than that in HER2^+^-type patients, and the SGS of epithelial cells in HER2^+^-type patients was greater than that in ER^+^-type patients (Figure 5C), which is consistent with the bulk sample data. In addition, we selected the top 6 specific marker gene expressed by high SGS group cells for survival analysis in three bulk sample breast cancer data, and all of these genes were associated with a poor prognosis (Figure 5D). These results suggest that SGS is applicable to breast cancer classification and prognosis prediction based on scRNA-seq.

### Intercellular interactions

Cell communication analysis was performed on patients in the high and low SGS groups. More interactions were found in the high SGS group than in the low group (Figure 5E). In addition, the two groups had different types of intercellular interactions. Multiple intercellular interactions were stronger in the high SGS group vs. the low group (Figure 5F). Many signaling pathways, such as WNT, VEGF, and TGF-β, reported to be associated with the development of cancer [17–19], were found to be up-regulated in the high SGS group (Figure 5G). Cellular interactions in which the recipient cells are tumor epithelial cells were screened out. We found that the signaling differences between the two groups were mainly in endothelial cells and tumor-associated fibroblasts (Supplementary Figure 4). Endothelial cells and fibroblasts in the high SGS group expressed high amounts of collagen and released some growth factors such as IGF1 and FGF7, as well as NOTCH ligands, including JAG1 and DLL4. NOTCH signaling has been reported to be associated with the maintenance of stemness in breast cancer stem cells [20].

### Correlation of the SGS with macrophage infiltration

After differentiating patient prognosis at the single-cell level, further analysis was performed to characterize the immune microenvironment of patients. Macrophages plays an important role in tumor progression. The proportion of M1 and M2 co-expressing macrophage was found to be higher in the high SGS group than in the low SGS group. The proportion of macrophages not expressing either M1or M2 marker genes was found to be lower in the high SGS group than in the low SGS group. The proportion of M1-high, M2-low or M1-low, M2-high macrophages was comparable in both groups (Figure 5H).

### SGS application for clinical research

**Figure 6:**
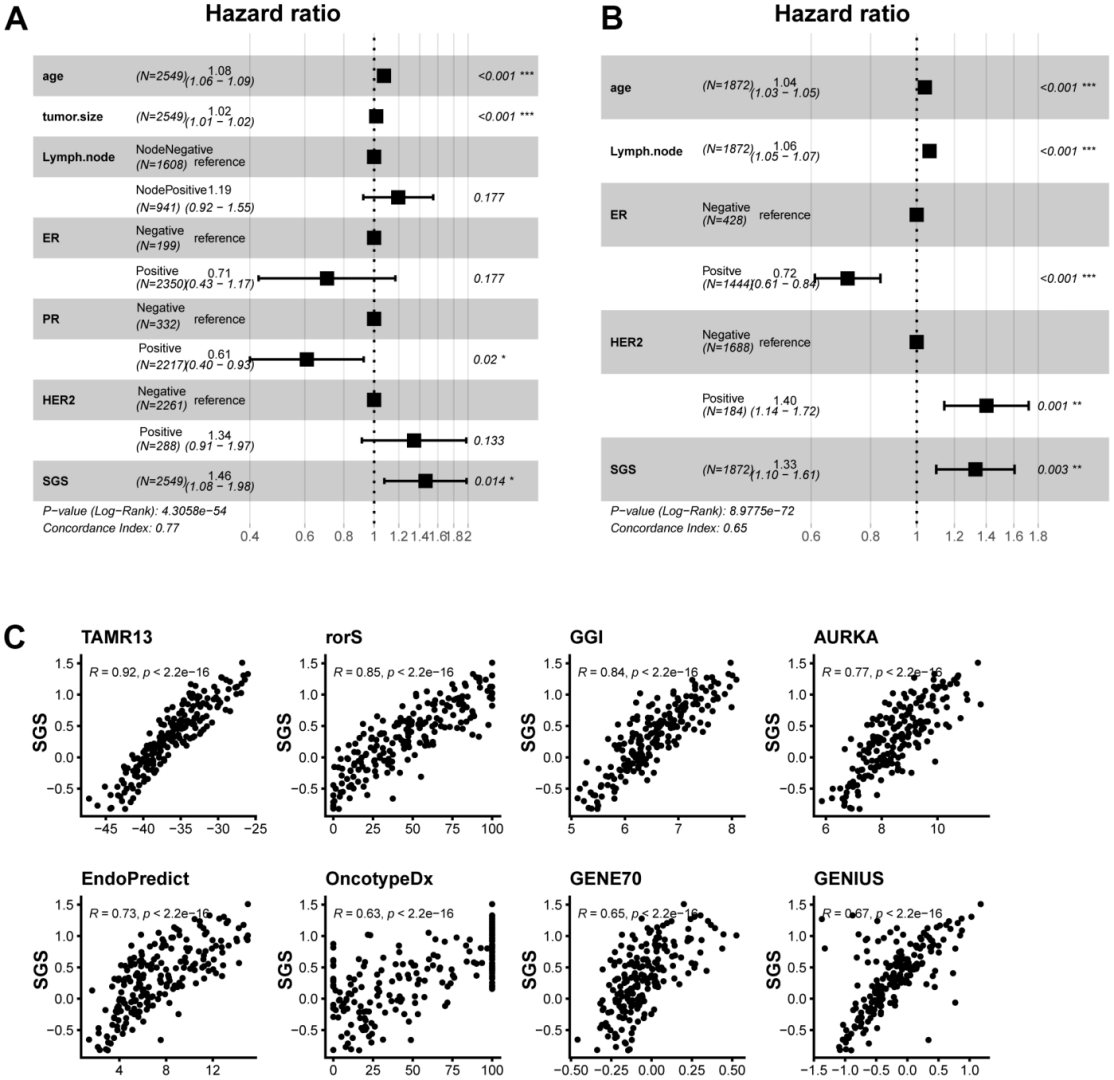
(**A**, **B**) Forest plot of multifactorial cox regression of clinical information and SGS in the SCAN-B (**A**) and TCGA (**B**) datasets. (**C**) Scatter plot of correlation between SGS and some common prognostic calculations.

One-way cox regression analyses were performed in several datasets to determine whether SGS could be used as an independent prognostic factor in the clinic. The results suggested that SGS could be used as a risk factor and was associated with prognosis. Next, multivariate Cox regression analysis of SGS with clinical information were performed in SCAN-B dataset and METABRIC dataset. The results were consistent with the univariate analysis results (Figure 6A, 6B). These results indicate that the SGS can be used as an independent prognostic factor in clinical studies. Some clinical methods for calculating breast cancer prognosis that are widely used and clinically validated, such as GENE70 and genomic grade index (GGI), were compared with the SGS (Figure 6C). The SGS was found to have a strong correlation with these algorithms. Our results demonstrate that SGS have important clinical application value.

## DISCUSSION

In this study, BC patient samples were downloaded from the TCGA-BRCA and METABRIC datasets. We used ssGSEA to calculate the NES for each sample and scored individual sample to obtain a stable SGS. No normalization process was required. Our results indicated that the SGS was a stable and reliable prognostic tool that was significantly associated with overall survival (OS) for BC patients. Currently, several breast cancer prognosis-prediction models are available. Generally, these tools have been developed based on the selection of prognostic genes based on bulk RNA-seq data and internal validation [21–23]. Typically, internal validation is not sufficient to fully validate a model. Compared to these tools, the SGS is applicable to single cell data and is a powerful tool for predicting breast cancer prognosis at the single-cell level. Moreover, we performed external validation using two independent datasets to evaluate the prediction accuracy of the prognosis tool instead of internal validation, thus making the results more reliable and useful. These factors give SGS an advantage over other tools.

We perform GSEA to detect the difference between groups. At the bulk level, the enriched pathways in the two groups presented a very different landscape. For example, E2F target and G2M checkpoint genes were significantly up-regulated in the high SGS group. This suggests that the cancer cells of BC patients in the high SGS group may have a higher proliferation capacity and higher malignant potential [24, 25]. Some pathways, such as mTOR and MYC pathways, were also found to be significantly upregulated in the high SGS group. These pathways have been reported to be associated with breast cancer malignancy [26, 27]. At the single cell level, cell communication analysis also suggested that the signaling pathways such as WNT, VEGF, and TGF-β, which are widely known associated with tumor malignancy, were up-regulated in the high SGS group [17–19] NOTCH signaling has also been reported to be associated with the maintenance of stemness in breast cancer stem cells [20]. Our findings may be correlate with high tumor progression and metastasis. These results may explain the hyperactivated state of tumor progression and metastasis in patients with a high SGS.

The tumour microenvironment (TME) plays a critical role in cancer growth and metastasis [28]. Recent studies have revealed that TME in breast cancer patients is highly heterogeneous and that the heterogeneity of the TME may also indirectly contribute to poor survival status. Specific TME characteristics have been implicated in the development of treatment resistance [29–31]. To understand the differences in immune patterns between the two groups of patients, we performed immune infiltration analysis and found differences in the abundance of various immune cells, such as macrophages, Tfh, and mast cells, between the two groups. Current studies have found that tertiary lymphoid structures (TLSs) in the TME correlate with patient survival in multiple cancer types and that Tfh, Tregs and other TLS cells play a regulatory role in the development of cancer [32–35]. Gobert et al. reported that Tregs in breast cancer can selectively aggregate via CCR4, preventing effector T-cell activation and ultimately leading to immune escape and tumor progression, indicating that Tregs infiltration may associated with a poor prognosis [36]. Our results of immune infiltration suggest that the difference in prognosis between the two groups may be due to the difference in immune cell compositions in the TME, as the TME can be immunosuppressive and promote immune escape and tumor progression.

Typically, the phenotypes of macrophages are classified into M1 and M2 types; M1 macrophages display tumoricidal activity, while M2 macrophages promote tumor progression [37]. In the tumor microenvironment of breast cancer patients, M2-type macrophages predominate [38, 39]. With the rise of single-cell sequencing technologies, Zhang Zeming et al. proposed that macrophage cannot be simply classified into M1 and M2 types [40]. Single-cell sequencing confirmed that co-expression of M1 and M2 markers in individual cells. Recent work by Fanjia et al. supports this view [41]. Therefore, there is still much unknown about the relationship between macrophage polarization and cancer prognosis. Our study also found that the proportion of M1 and M2 marker-co-expressed macrophage in the TME was much higher in the high SGS group than in the low SGS group. This implies that the co-expression of M1 and M2 macrophage markers may be pro-cancer phenotype in breast cancer. This study differs from previous studies and may provide new insight into the development of breast cancer immunotherapy in the future.

In conclusion, SGS is closely related to prognosis. Our findings suggest the feasibility of the clinical application of SGS (Figure 6A, 6B). Moreover, SGS is a marker associated with immune infiltration, providing new ideas for immunotherapy in BC patients. Several study limitations need to be acknowledged. First, the data in our study were obtained from public databases that were not generated by us, and the quality of the data could not be well appraised. Second, our study used the median of the SGS to classify breast cancer samples into the high SGS group and low SGS group. More precise cut-off points may exist for classifying BC patients. Third, although immune cell types in the TME of patients between different groups were found to be significant different by immune infiltration analysis, the biological mechanisms behind these immune cell types are unclear. More studies are needed to specify the role of the immune contexture in breast cancer.

## MATERIALS AND METHODS

### Datasets acquisition and pre-processing

TCGA-BRCA data were downloaded using TCGAbiolinks (TCGA-BRCA, n=1049) [42] and transformed to transcripts per million (TPM). Then, we used the ‘removeBatchEffect’ function in the limma R package [43] to integrate the TCGA-BRCA and METABRIC breast cancer datasets.

SCAN-B(GSE96058) data was obtained using the R package GEOquary. The other microarray datasets (including GSE1456-GPL96 (n=159), GSE16446 (n = 107), GSE20685 (n = 327), GSE20711 (n = 88), GSE42568 (n = 104), GSE45255 (n=134), GSE58812 (n=107), GSE65194 (n=130), GSE69031 (n=129), GSE7390 (n=198)) were also obtained from the Gene Expression Omnibus (GEO, http://www.ncbi.nlm.nih.gov/geo/) database. Raw data (CEL files) were normalized by robust multichip average (RMA) using the affy R package [44]. Subsequently, probe annotations were performed using the idmap1 R package. For each gene, the probeset with the highest expression was kept. Batch effects were removed using the limma package.

GSE161529 is the single cell dataset of normal breast and breast cancer with the largest sample size at present [16]. Seurat package was used for quality control [45], leaving approximately 150 thousand high-quality cells. Then, the batch effects were removed by harmony R package [46].

### SGS construction and validation

Thirty-six stemness gene sets were recruited from the website: StemChecker (http://stemchecker.sysbiolab.eu/), which contains many stemness-associated genes of murine and human origin [47]. Then, ssGSEA [48] was implemented to quantitatively elucidate the NES of the 36 stemness gene sets in each BC sample. The NES was designed to estimate the immune infiltration level of an stemness gene set for each sample. Univariable Cox proportional hazards regression analysis was used to assess the association of NES values with OS in the breast cancer cohort datasets and screen OS-associated gene sets. Gene sets with *P*-values less than 0.05 were retained. Finally, we determined 18 OS-associated gene sets (shown in the results). The hazard ratio (HR) was computed by univariate Cox regression analysis. An HR greater than 1 indicated gene sets whose Cox coefficient was negative, while an HR less than 1 indicated gene sets whose Cox coefficient was positive. Based on the NES value and HR, the SGS for each breast cancer sample was calculated as follows:


$$SGS=\sum_{i=1}^{n}NES-\sum_{j=1}^{m}NES$$

Where NES*_i_* is the NES with an HR more than 1 and NES*_j_* is the NES with an HR less than 1.

### Tumor microenvironment infiltration imputation

The CIBERSORTx deconvolution algorithm could robustly quantify the relative proportion of various cell types through gene expression profiling [49]. It also provides 22 kinds of processed immune cells that can be directly used in the algorithm. We also used ssGSEA to calculate the infiltration scores of 28 kinds of stromal cells [50].

### Chemotherapy sensitivity predictions

The half-maximum inhibitory concentration (IC50) values of several drugs in each BC sample were computed for the prediction of chemical sensitivity via the oncoPredict R package [51], and the prediction accuracy was evaluated by 10-fold cross-validation based on the Genomics of Drug Sensitivity in Cancer (GDSC, https://www.cancerrxgene.org/) training set [52].

### Differential gene expression analyses and gene set enrichment analyses

Bulk samples’ differential gene expression analysis was performed using R package limma [53]. Single-cell differential expression analysis was determined using the FindMarkers function from the R package Seurat [45], with the logFC threshold parameter set to 0.1.

GSEA, KEGG pathway analysis and Gene Ontology (GO) analysis was performed using the clusterProfiler R package [54]. Cancer hallmark gene set were downloaded from msigdbr R package. Univariate Cox regression, multivariate Cox regression analyses, and Kaplan–Meier survival analysis were conducted by the survminer and survival R packages.

The genefu R package was used to classify all samples into PAM50 molecular subtypes [55]. Single-cell clustering was performed using the Seurat R package [45]. After cell clustering, specific marker genes were used to define each cell population. For the cell type annotation of a single cell subpopulation, the R package SingleR was used [56], thus distinguishing the myeloid cell population into three cell types, monocytes, macrophage, and dendritic cells (DCs). Cell-cell communication analysis was carried out using the CellChat R package [57].

### Statistical analyses

All statistical analyses were performed in R software (v4.1.0). The Wilcoxon test was used for pairwise comparisons between two groups, and the Kruskal–Wallis test was used for multiple group comparisons. The Kaplan–Meier method and log-rank test were performed for survival analysis. The optimal cutoff value of the stemness-risk score was determined by the “surv_cutpoint” function of the survminer R package (v0.4.6). A P value < 0.05 was considered to indicate statistical significance.

## Supplementary Material

Supplementary Figures
