# Supplementary material for: Construction of stemness gene score by bulk and single-cell transcriptome to characterize the prognosis of breast cancer
Source: Aging (Albany NY). 2023 Aug 18;15(16):8185–203. doi: 10.18632/aging.204963 (PMC10496995; doi:10.18632/aging.204963)
Supplement: Supplementary Figures [file aging-15-204963-s001.pdf]

SUPPLEMENTARY FIGURES

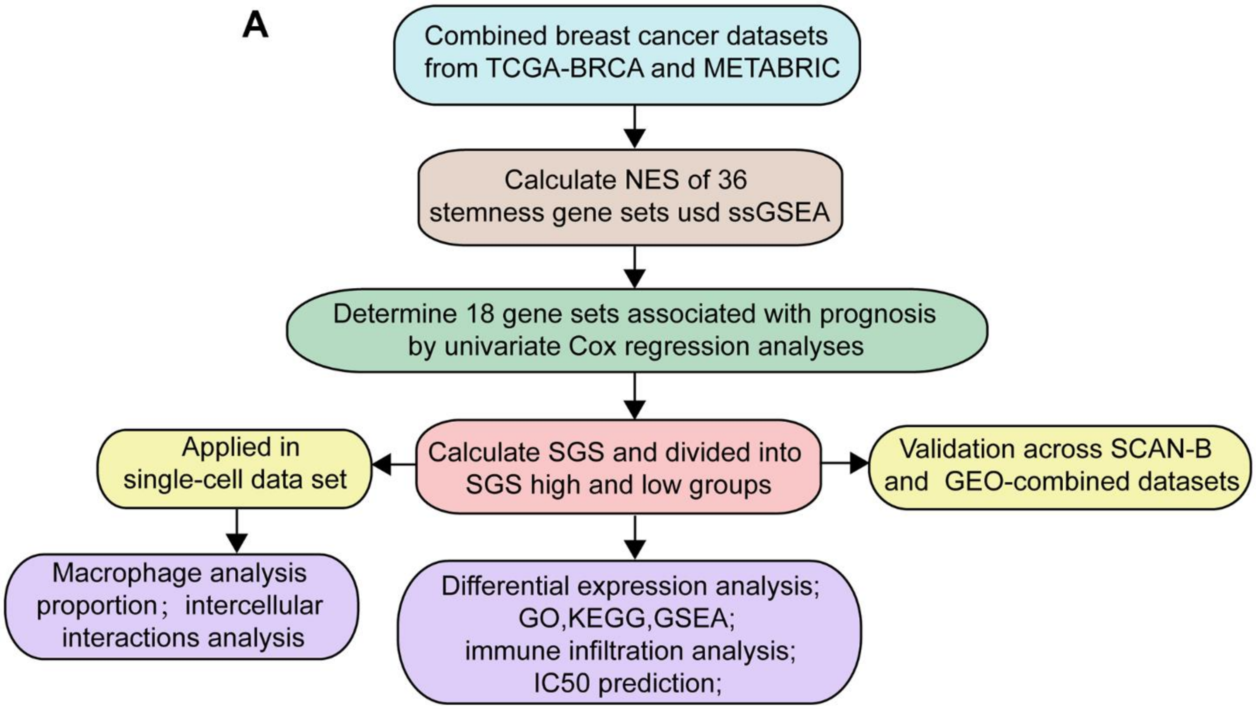

Supplementary Figure 1. Workflow of this study.

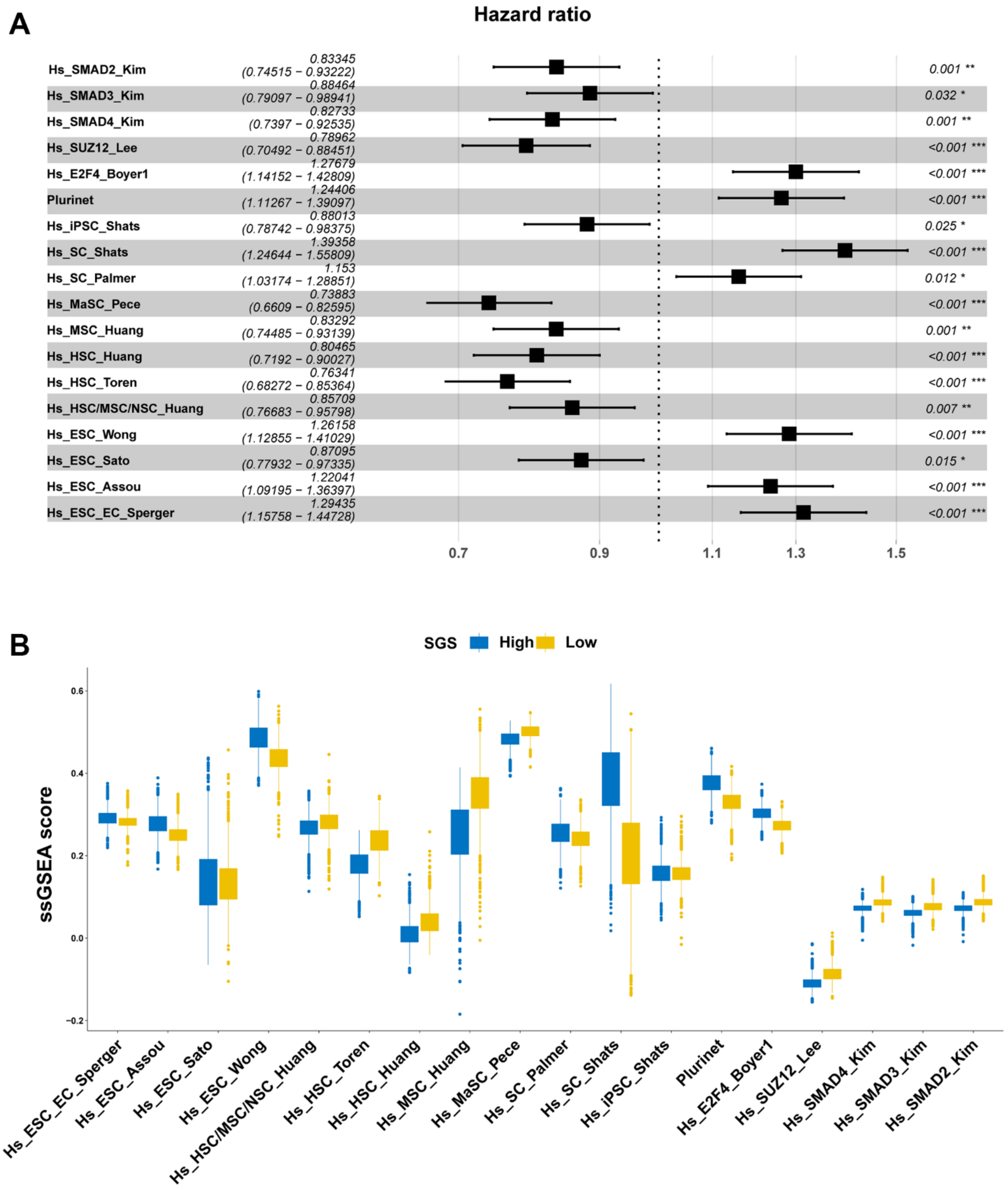

Supplementary Figure 2. (**A**) Univariate COX regression analysis of ssGSEA scores and OS for prognosis-related stemness gene sets. (**B**) ssGSEA scores of 18 prognosis-associated stemness gene sets.

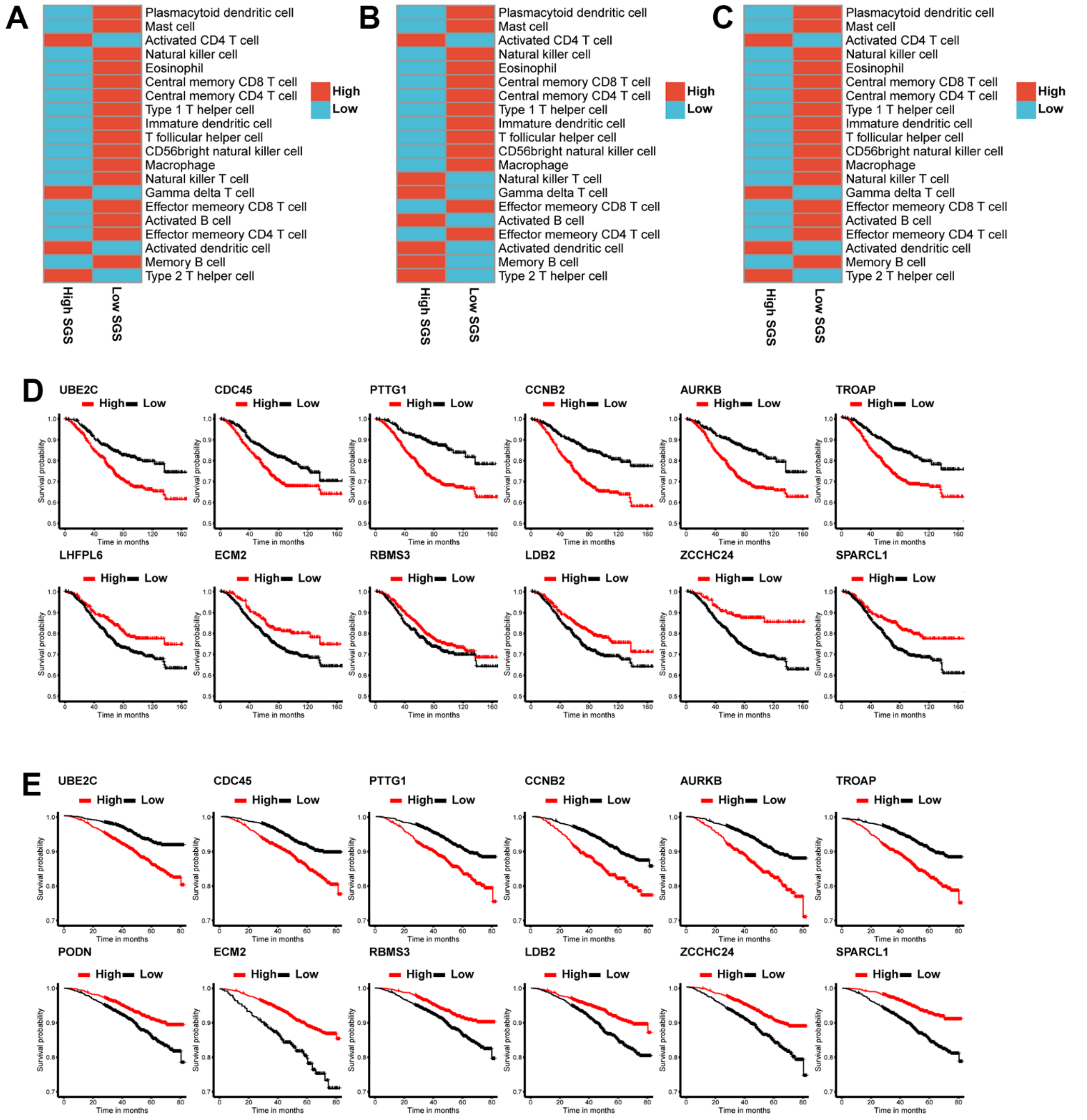

Supplementary Figure 3. (**A**–**C**) Immune infiltration analysis of TCGA and METABRIC combined dataset (**A**) GEO-combined dataset (**B**) and SCAN-B dataset (**C**). (**D**, **E**) KM survival analysis of top6 up-and down-regulated genes in the GEO -combined dataset (**D**) and SCAN-B dataset (**E**).

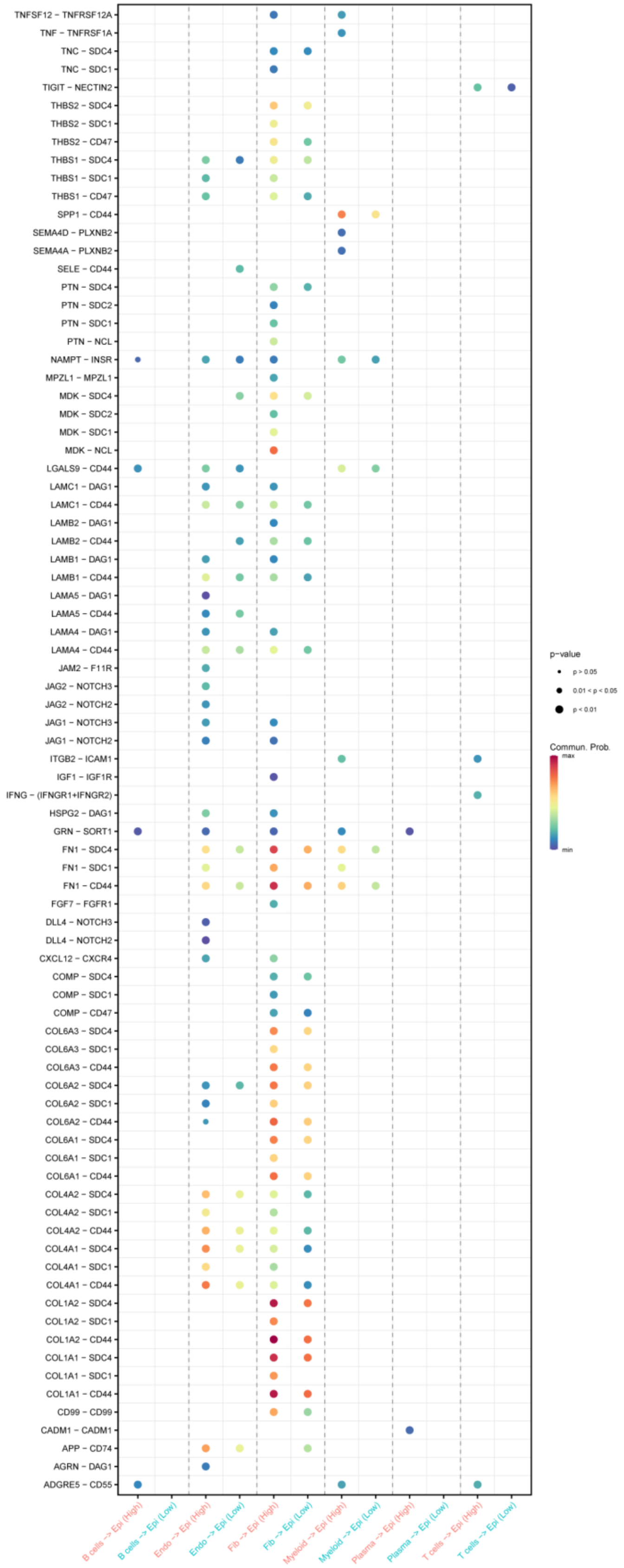

Supplementary Figure 4. Dot plot with ligand-receptor interactions between stromal cells and endothelial cells. Point size indicates P value. Color indicates the strength of the interaction.
